# A Sustainable Strategy to Prevent Misuse of Antibiotics for Acute Respiratory Infections

**DOI:** 10.1371/journal.pone.0051147

**Published:** 2012-12-12

**Authors:** Gail B. Rattinger, C. Daniel Mullins, Ilene H. Zuckerman, Eberechukwu Onukwugha, Loreen D. Walker, Adi Gundlapalli, Matthew Samore, Sylvain DeLisle

**Affiliations:** 1 Veterans Affairs Maryland Health Care System, Baltimore, Maryland, United States of America; 2 School of Pharmacy, University of Maryland, Baltimore, Maryland, United States of America; 3 School of Pharmacy, Fairleigh Dickinson University, Florham Park, New Jersey, United States of America; 4 Veterans Affairs Salt Lake City Health Care System, Salt Lake City, Utah, United States of America; 5 School of Medicine, University of Utah, Salt Lake City, Utah, United States of America; 6 School of Medicine, University of Maryland, Maryland, United States of America; University of Chieti, Italy

## Abstract

**Backgrounds:**

Over 50% of antibiotics prescriptions are for outpatients with acute respiratory infections (ARI). Many of them are not needed and thus contribute both avoidable adverse events and pressures toward the development of bacterial resistance. Could a clinical decision support system (CDSS), interposed at the time of electronic prescription, adjust antibiotics utilization toward consensus treatment guidelines for ARI?

**Methods:**

This is a retrospective comparison of pre- (2002) and post-intervention (2003–2006) periods at two comprehensive health care systems (intervention and control). The intervention was a CDSS that targeted fluoroquinolone and azithromycin; other antibiotics remained unrestricted. 7000 outpatients visits flagged by an ARI case-finding algorithm were reviewed for congruence with the guidelines (antibiotic prescribed-when-warranted or not-prescribed-when-unwarranted).

**Results:**

3831 patients satisfied the case definitions for one or more ARI: pneumonia (537), bronchitis (2931), sinusitis (717) and non-specific ARI (145). All patients with pneumonia received antibiotics. The relative risk (RR) of congruent prescribing was 2.57 (95% CI = (1.865 to 3.540) in favor of the intervention site for the antibiotics targeted by the CDSS; congruence did not change for other antibiotics (adjusted RR = 1.18 (95% CI = (0.691 to 2.011)). The proportion of unwarranted prescriptions of the targeted antibiotics decreased from 22% to 3%, pre vs. post-intervention (p<0.0001).

**Conclusions:**

A CDSS interposed at the time of e-prescription nearly extinguished unwarranted use targeted antibiotics for ARI for four years. This intervention highlights a path toward sustainable antibiotics stewardship for outpatients with ARI.

## Introduction

Microorganisms resistant to antibiotics increase the mortality, morbidity and costs of infections. Without a drug development infrastructure that can keep pace with the rapidly evolving resistance mechanisms, these organisms are expected to threaten public health for years to come.

Because exposure to antibiotics is a key promoter of bacterial resistance [1], efforts aimed at minimizing the unnecessary use of antibiotics could slow the rate at which resistance emerges. To date, such efforts have largely targeted uncomplicated acute respiratory infections (ARI) [2], a rubric that includes outpatient conditions for which antibiotics are routinely over-prescribed, namely acute bronchitis, acute sinusitis, acute pharyngitis and nonspecific upper respiratory tract infection (URI) [3]. Traditional educational methods aimed at health care providers can reduce unwarranted antibiotics use, but their effect tend to be modest [2] and ephemeral [4].

In this work, we re-engineered pharmacy processes to interpose a clinical decision support systems (CDSS) at the time of order entry for selected antibiotics [5]. The hypothesis was that integrating electronic tools within the natural flow of care could sustainably counteract a persistent form of unwarranted drug use.

## Methods

This is a retrospective, observational study designed to assess the effect of a CDSS on congruence of antibiotics prescribing with widely endorsed ARI treatment guidelines [6]. The study period ranged from January 2002 to December 2006. The CDSS intervention started in January 2003 at the Veterans Affairs (VA) Maryland Health Care System and continued through the end of the study period. The VA Salt Lake City Health Care System served as a control site. The CDSS targeted azithromycin and gatifloxacin, which had been the most frequently prescribed antibiotics in outpatients with uncomplicated ARI at the intervention site. All other outpatient antibiotics remained unrestricted.

### Ethics Statement

The Institutional Review Boards of the participating VA health systems, the University of Maryland and the University of Utah, approved the study. The study was granted a waiver of consent as risks were limited to information confidentiality and it involved a large number of participants.

### Description of the Intervention

#### Setting and process engineering

The CDSS intervention was part of a larger quality improvement initiative that targeted 26 medications and was used by at least 1379 unique providers during the study period. The CDSS: 1) deployed drug-specific guideline recommendations as clickable choices during order entry; 2) mined the electronic medical record (EMR) for patient/context specific information; and 3) based on what the provider had clicked on, issued a note documenting the rationale for drug use. The providers could then accept or modify this note before committing it to the EMR. The presence of this note was verified by pharmacy, but its content was not routinely audited.

#### Antibiotics CDSS

The azithromycin and gatifloxacin CDSS included treatment paths for the following diseases: community-acquired pneumonia, acute bronchitis, acute sinusitis, non-specific upper respiratory infection (URI) and exacerbations of chronic obstructive pulmonary disease (COPD). An “Other” path provided access to either drug for provider-supplied indications.

For the community-acquired pneumonia path, providers clicked on the diagnostic elements that raised their index of suspicion for pneumonia and were then led to a prescription. For the acute bronchitis [7], acute sinusitis [8] and non-specific URI [9] paths, the software first verified that the clinical condition matched the guideline’s case definition and then sought to identify clinical circumstances where antibiotics could be warranted. This included acute bronchitis patients with abnormal vital signs (temperature >38°C or respiratory rate >22 breath per minute or pulse >100) or with clinical signs of lung consolidation, and acute sinusitis patients who were febrile (temperature >38°C) or who had severe or persistent (7 days or more) symptoms. Under those circumstances, the CDSS led to an antibiotic prescription. For cases with acute bronchitis and sinusitis where guidelines suggested that antibiotics could be safely withheld, and for all cases with non-specific URI, the software did not lead to a prescription. Instead, providers were advised on how to maintain patient satisfaction when withholding antibiotics. Providers reaching such an outcome but wishing to prescribe antibiotics anyway could override the system by using a path known to lead to a prescription (e.g. the “Other” path or the “Pneumonia” path) and then typing their own rationale for drug use in the CDSS-issued note. They were also free to prescribe antibiotics not targeted by the CDSS.

### Participants

A case-detection algorithm previously found to identify 76% of patients with an influenza-like illness [10] was applied to EMR-derived relational databases. Outpatient visits during the study period (n = 4.1 million) were flagged if providers either assigned an ARI-related diagnostic code [10] or prescribed a cough suppressant, and if the clinical note documented at least two ARI symptoms, as assessed by automated text analysis of [10].

### Record Review

With the exception of notes generated by the CDSS itself, all free-text EMR entries on the day of flagged visits were manually abstracted for data elements needed to assign ARI diagnoses and treatment. Reviewers cross-validated 10% of each other’s work; conflicts were resolved through arbitration with a pulmonary medicine specialist. Inter-rater reliability was determined using 15% of the total sample for the “cough”, “sputum production”, “cough duration” and “sputum production duration” symptoms (kappa statistics = 0.80, 0.87, 0.87 and 0.87 respectively at the intervention site and 0.86, 0.79, 0.91, and 0.92 respectively at the control site). Structured EMR data elements, such as prescriptions and vital signs, were extracted from the EMR [10] and later appended to the review database.

### Exclusion, Diagnostic and Treatment Criteria

We reviewed all of the records flagged by the case-detection algorithm at the intervention site. Based on preliminary results and published effect sizes [2], we calculated that abstracting 2,000 flagged outpatient encounters would have 80% power to detect a 10% change in congruence at the control site.

Of the 7000 visits manually reviewed, 3169 were excluded according to pre-defined criteria: 1) not an outpatient (n = 141); 2) not an ARI (n = 855); 3) not an in-person, initial visit for a given ARI episode (no prior ARI visit within 3 weeks) (n = 1093); 4) prior ARI episode(s) during the study period i.e. patients were used only once (n = 140); 5) stated diagnosis of COPD, whether or not the visit was related to an exacerbation of this disease (n = 501); 6) acute pharyngitis as the only ARI diagnosis (n = 431).

A visit was labeled pneumonia if a provider note listed this diagnosis as likely. Other ARI case definitions and conditions justifying antibiotics matched that of the guidelines (Table 1) [7], [8], [9], [11]. Visits for conditions other than pneumonia or non-specific URI could be labeled with more than one ARI diagnoses. Antibiotics were categorized as “warranted” if they were justified for at least one ARI diagnosis. We defined an ARI visit as “congruent” with the guidelines if an antibiotic was either prescribed or withheld in accordance with the criteria outlined in Table 1.

**Table 1:** Diagnostic and treatment criteria for ARI.

ARI Condition	Diagnostic Criteria	Antibiotic Treatment Criteria
Pneumonia	Clinician’s documented diagnostic impression	Antibiotics always warranted
Acute Bronchitis	1) Acute cough (productive or not) 2) Cough duration <21 days	Antibiotics not warranted
Pharyngitis	1) Sore throat 2) Erythematous throat	At least three of the following four symptoms/signs: 1) History of fever or temperature >100.4°F (38°C); 2) Tonsillar exudate; 3) Tender anterior cervical lymphadenopathy; 4) Absence of cough
Sinusitis	1) Purulent nasal discharge2) Facial or sinus pain 3) Sinus tenderness4) Productive cough	Severe symptoms or symptom duration ≥7 days including purulent nasal discharge/drainage AND (Maxillary facial or tooth (sinus) pain OR tenderness)
Non-Specific Acute Upper Respiratory Infection	1) Absence of a predominant sinus, pharyngeal or lower airway symptom 2) Nasal discharge 3) Sputum production from the throat	Antibiotics not warranted

### Statistical Analysis

Descriptive statistics and results from bivariate tests for association were generated for the study sample and informed the multivariable analyses. Multivariable logistic regression and difference-in-difference regression analyses using a Poisson distribution with a log link and modified Poisson approach to obtain robust standard error estimates [12] were developed to estimate the impact of the CDSS intervention on overall antibiotics prescribing congruence (SAS software v. 9.2, SAS Institute Inc. Cary, NC). The size of inference tests for covariates in all adjusted regression models was set *a priori* at 0.05. Patient-specific covariates were age, marital status at index visit date, sex and self-reported race/ethnicity (African-American/other).

## Results

### Characteristics of Study Patients and ARI Diagnoses

The study included 3831 unique patients with an initial visit for ARI. Patients were mostly older males (Table 2). The most common ARI diagnosis was acute bronchitis (76.5% of all ARI visits), followed by pharyngitis (40.7%), sinusitis (18.7%), pneumonia (14.0%) and non-specific URI (3.8%). More than one ARI diagnosis was found in 56.9% of ARI visits (Table 2).

**Table 2:** Demographics and Diagnoses.

Characteristics	Intervention Site N (%)	Control SiteN (%)
Sample Size	2669	1162
Sex		
Male	2439 (91.4)	1096 (94.32)
Female	230 (8.6)	66 (5.68)
Self-Reported Race		
African American	1775 (66.5)	18 (1.55)
White	601 (22.5)	697 (59.98)
Latino	17 (0.6)	41 (3.53)
Other	10 (0.4)	14 (1.20)
Missing	266 (10.0)	392 (33.73)
Marital Status		
Married	792 (29.7)	620 (53.36)
Unmarried	1877 (70.3)	542 (46.64)
Age at Encounter Date, years		
Mean	55.6 (13.9)	59.1 (15.55)
Median	53	58
Range	16–97	19–91
ARI Visits, by year		
* Pre - intervention*		
2002	373 (14.0)	344 (29.60)
*Post - intervention*		
2003	673 (25.2)	253 (21.77)
2004	481 (18.0)	185 (15.92)
2005	770 (28.9)	225 (19.36)
2006	372 (13.9)	155 (13.34)
ARI Visits, by Condition(s)		
Pneumonia	337 (12.6)	200 (17.2)
Bronchitis Only	941 (35.3)	292 (25.1)
Sinusitis Only	57 (2.1)	66 (5.7)
Bronchitis plus pharyngitis	918 (34.4)	281 (24.2)
Bronchitis plus sinusitis	124 (4.6)	109 (9.4)
Bronchitis plus pharyngitis plus sinusitis	139 (5.2)	127 (10.9)
inusitis plus pharyngitis	51 (1.9)	44 (3.8)
on-specific URI	102 (3.8)	43 (3.7)

Counts of ARI Patients and Visits by Characteristics and Sites over the whole study period (2002–2006, n = 3831).

### Effect of the CDSS on Warranted Use of Antibiotics

Of the 624 visits where antibiotics were warranted (Table 3), most were for pneumonia (n = 537, 86%). All pneumonia patients received antibiotics. Of the remaining 87 visits, 18 (20.7%) did not receive antibiotics.

**Table 3:** Antibiotic Prescriptions by Visits.

	Intervention Site				Control Site			
Antibiotics	Warranted		Unwarranted		Warranted		Unwarranted	
	2002	2003–6	2002	2003–6	2002	2003–6	2002	2003–6
Prescribed (Targeted)	26	225	55	45	48	111	34	100
Prescribed (Others)	13	110	84	583	23	50	51	148
Not Prescribed	0	7	195	1326	6	5	182	404
Total (3831)	39	342	334	1954	77	166	267	652

Number of ARI visits for which antibiotics were given (“Prescribed” rows) or withheld (“Not Prescribed” row) either in accordance with (“Warranted” columns) or against guideline recommendations (“Unwarranted” columns). Columns further separate the visits by 1) study site (Intervention vs. Control Site); 2) time periods (pre-intervention year (“2002”) vs. post-intervention years (“2003–6”)); and 3) by whether or not the antibiotics prescribed were those targeted by the intervention (“Targeted” vs. “Other”).

**Figure 1:**
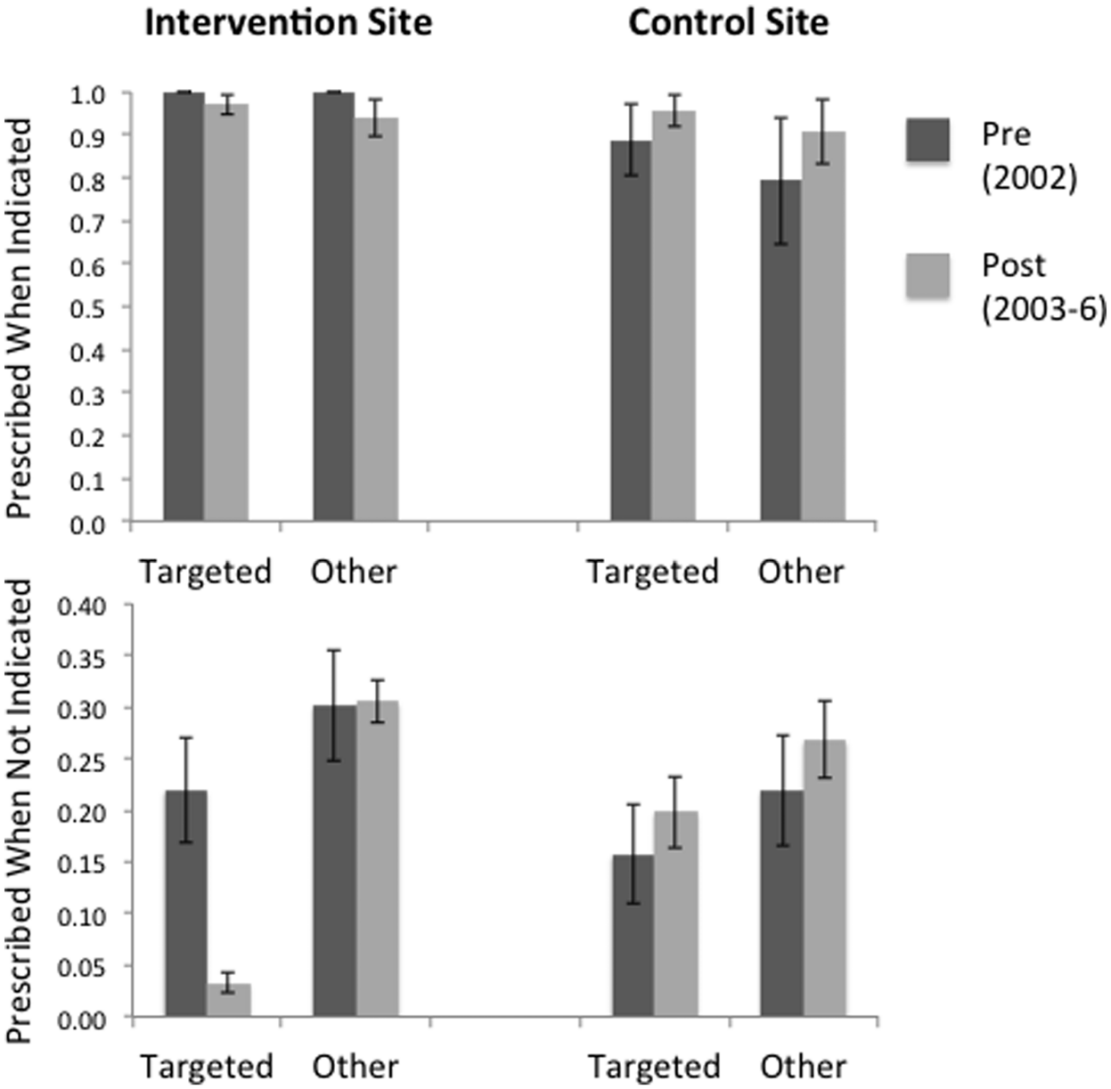
Effect of the CDSS on antibiotics use for ARI. Comparison of antibiotic utilization for ARI between the time periods before (dark bars) or after (light bars) introduction of the CDSS. Y-axis represents proportions of ARI visit where antibiotics were prescribed. For comparisons involving antibiotics targeted by the CDSS (dark and light bar pairs over the word “Targeted”), proportions are given by V_Targeted_/(V_Targeted_+V_NoAntibiotic_), where V_Targeted_ is the number of visits where targeted antibiotics were prescribed and V_NoAntibiotic_ the number of visits not issued antibiotics. For comparisons involving other antibiotics (dark and light bar pairs over the word “Other”), proportions are given by V_Other_/(V_Other_+V_NoAntibiotic_), where V_Other_ is the number of visits where Other antibiotics were prescribed. Upper panel only includes ARI visits where antibiotics were indicated; lower panel only includes those ARI visits where antibiotics were not indicated. Results for the intervention and the control sites are given on the left and right side of the figure, respectively. Note that proportions of visits where antibiotics were prescribed did not change pre vs. post-intervention, except for a decrease in Targeted antibiotics use at the intervention site.

Figure 1 compares pre- vs. post-intervention proportions of ARI visits prescribed an antibiotic. The upper panel focuses exclusively on ARI visits where antibiotics were warranted and illustrates that providers prescribed antibiotics in a high proportion of such visits. These proportions did not change appreciably from the pre- to the post-intervention periods at both study sites. The targeted antibiotics remained the most prescribed of the warranted antibiotics at both study sites (66.7% vs. 67.2% (Maryland) and 67.6% vs. 68.9% (Utah) of total warranted prescriptions, pre- vs. post-intervention).

### Effect of the CDSS on Unwarranted Use of Antibiotics

The majority of ARI visits did not include documentation supporting the use of antibiotics (n = 3207 or 83.7% of total ARI visits, Table 3). For the drugs targeted by the CDSS, the proportion of unwarranted prescriptions decreased from 22% to 3.3% (p<0.0001) of visits, pre- to post-CDSS (Fig. 1, lower panel). The equivalent proportion remained unchanged for the Other antibiotics at the intervention site (30.1% to 30.5%), or for both the Targeted (16% vs. 20%) and the Other (22% vs. 27%) antibiotics at the control site.

### Effect of CDSS on Overall Antibiotic Prescribing Congruence with the ARI Guidelines

The proportion of total ARI visits where antibiotics use was congruent with the guidelines increased from the pre- to the post-intervention periods at the intervention site (from 0.63 to 0.72, p = 0.0001), but was unchanged at the control site (from 0.74 to 0.69, p = 0.10). At the intervention site, congruence increased in the first post-intervention year (0.72 (95% CI = (0.68, 0.75) in 2003) and remained stable afterwards (0.73 (95% CI = (0.69, 0.77) in 2004, 0.72 (95% CI = (0.69, 0.75) in 2005, and 0.73 (95% CI = (0.69, 0.78) in 2006). Prevalence ratios of antibiotics prescribing congruence were obtained by site and by year through adjusted multivariable logistic regression models. Adjusted multivariable difference-in-difference models between the two study sites, post- vs. pre-intervention periods, revealed a relative risk (RR) of a congruent prescription to be 1.24 (95% CI = (1.110, 1.391)), in favor of the intervention site. The impact of the intervention was found to be greatest for the targeted antibiotics (RR = 2.57; 95% CI = (1.865, 3.540)). Prescribing congruence for antibiotics not targeted by the intervention was unchanged (adjusted RR = 1.18; 95% CI = (0.691, 2.011)). No change was detected in congruence when no antibiotics were prescribed (adjusted RR = 0.99; 95% CI = (0.990, 1.00)). Favorable adjusted relative risks persisted for the sub-group of patients without pneumonia (n = 3294, RR = 1.27, 95% CI = (1.112, 1.457)) and in patients with acute bronchitis as their only ARI diagnosis (n = 1233, RR = 1.32; 95% CI = (1.042, 1.678)).

## Discussion

In this work, a CDSS interposed treatment guidelines at the time of electronic order entry for antibiotics frequently used for outpatients with ARI. We report that the indicated use of the two antibiotics targeted by the CDSS, azithromycin and gatifloxacin, remained undiminished, but that their unnecessary use for ARI was curtailed for a 4-year period. This outcome was not observed for antibiotics not subject to the CDSS at the intervention and at the control sites.

The strengths of our study include the long duration of the intervention, the large sample size, and explicit case definitions and treatment criteria. Statistical comparisons could be made not only across the pre vs. post intervention time period, but also between the targeted and non-targeted antibiotics. Because pre- and post-intervention data was available from the intervention and the control site, we could also use a quasi-experimental difference-in differences approach to control for factors other than the CDSS that could be contributing to time-dependent changes in congruence to ARI antibiotics guidelines. In absolute terms, the overall 9.5% post-intervention decline in unwarranted antibiotic use for ARI was consistent with the 9.7% median reduction observed in 30 conventional intervention trials reviewed by Ranji et al. [2]. What was exceptional is that we could attribute this decline almost entirely to the only two antibiotics targeted by the CDSS. For these antibiotics, CDSS filtered utilization apparently as intended: azithromycin and gatifloxacin remained the most popular antibiotics when indicated for ARI, but were seldom used when not needed.

From a safety and tolerability standpoint, providers must be allowed to override the recommendations of a CDSS. This design requirement could have allowed providers to bypass the chief aim of the intervention, which was to convince them not to prescribe antibiotics unnecessarily for ARI. In a first scenario, they could have redirected ingrained misutilization to antibiotics not subject to the CDSS. Had this been the only effect of the intervention, we estimate that the proportion of ARI visits where antibiotics were not warranted but where agents other than azithromycin and gatifloxacin were prescribed should have risen above 50% in the post-intervention period. Because this proportion remained unchanged at 30%, our data argue that the CDSS did not merely shunt misutilization toward alternative, unrestricted drugs. In a second scenario, providers could have assigned the diagnosis of “pneumonia” more liberally, thereby seemingly justifying antibiotics that, in fact, were not indicated. Had providers used this tactic to justify all unwarranted prescription of the targeted agents, the proportion of all ARI visits where the targeted antibiotics were prescribed would have remained unchanged. In reality, this proportion decreased from 21.7% at baseline to 11.8% post-intervention. Providing further reassurance that outcomes were not due to systematic “gaming” of the process, subgroup analyses that either excluded patients with a pneumonia diagnosis or that included only patients whose sole diagnosis was acute bronchitis yielded findings comparable to those found in the full cohort. Overall, and even though unintended actions such as those outlined in the above scenarios could have occurred more than occasionally, our data suggest that the main effect of the CDSS was to extinguish unneeded prescriptions of the targeted agents.

Many factors could limit the generalizability of our results. The study did not employ a randomized allocation process, leaving it susceptible to well-described biases [13]. The intervention was implemented at only one site, in the favorable context of a comprehensive health care system whose providers were familiar with prescription-based CDSS. As the difference in initial guideline-congruent prescribing between our two study sites illustrates, antibiotic prescribing may vary widely between sites [14], [15], [16]. Thus, a process similar to the one described in this study may need adjustments, require significant provider education and yet yield different outcomes when implemented elsewhere. The retrospective data collection meant that symptoms and signs were assumed to be absent if they were not documented in the medical record. Thus, ARI episodes could have been missed or mislabeled, along with the rationale justifying the use of antibiotics. Although prior studies aimed at improving antibiotics utilization have returned similar results in VA and non-VA environments [17], our study population was mostly male and did not include pediatric age groups or patients with COPD. By focusing on initial visits for ARI, the study also did not address ARI patients returning to clinics with unabated or worsening ARI symptoms. Because multiple practitioners, from medical students to attending physicians, were often involved in assessing patients, we could not formally control for potential predictors of prescribing congruence such as individual practitioners’ prior practices, level of experience and exposure to the CDSS. These limitations represent design opportunities for future, hopefully prospective multisite studies aimed at determining how similar interventions based would fare when extended to more antibiotics or to other health care settings.

The rate at which antibiotics are inappropriately used for ARI remains high but has been decreasing for more than a decade [18], [19], [20]. This decline, however, exhibits unfavorable features in an aging population: it has been concentrated in the pediatric population and may not be occurring at all in patients 50 years or older [21]. The decline has also not affected all antibiotics equally, with ARI-related utilization of azithromycin and fluoroquinolones actually increasing [21]. Thus, there is a continued need to develop better methods to improve ARI-related antibiotics use. CDSS have long proven capable to change prescribing practices, with most published applications directed at improving drug dosing [22] and safety monitoring [23], [24]. CDSS have also shown the potential to improve utilization of antibiotics, particularly in the hospital environment [25], [26]. To date, however, examples of demonstrated utility in the outpatient arena remain scarce [27], [28]. In the only study that included adults with ARI [28], providers were given the opportunity to consult a stand-alone CDSS in order to receive patient-specific recommendations. Our intervention is distinctive in that our CDSS was neither stand-alone nor optional. Instead, the CDSS was incorporated into a comprehensive EMR and interposed in the normal workflow leading to an e-prescription. The CDSS also did more than issue recommendations: it either did or did not lead to order entry and recorded the prescribing rationale thereby imparting at least the possibility of future accountability to a process that was otherwise operating as an honor system. These features, which had been associated with CDSS effectiveness in other domains [29], [30], were meant for the intervention to be stronger than what had previously been attempted for antibiotics stewardship purposes [5]. Yet, the path illustrated here did not include the vigorous traditional approaches associated with larger effect sizes in past trials i.e. one-on-one interactions, concurrent order reviews or practice audits [2], [31]. Our results therefore raise the hope that, at least for outpatients with ARI, counteracting antibiotic abuse may not require time-consuming, ultimately unsustainable activities.

From a disease-management standpoint, this CDSS intervention stood at a disadvantage because it targeted only a minority of the agents that could be used to treat ARIs. The system could nevertheless have effected large changes in overall guideline-congruent prescribing for ARI if it had fostered the transmission of information from providers to providers. Two lines of reasoning suggest that extra-CDSS educational transmission was not a major outcome of this intervention: 1) congruence gains were attributable to improved utilization of the CDSS-targeted antibiotics only; and 2) those gains were realized in the first post-intervention year and did not further increase afterwards. Thus, prescribing congruence did not exhibit the gradual expansion that would have been expected from an increasing the proportion of providers familiar with the ARI guidelines. Whether or not these outcomes are particular to a teaching institution with rapid housestaff turnover, they serve as a reminder that much more work will be required before we know how to best design, target and integrate prescription-based interventions to optimize the overall management of ARI.
